# *EBP1* Is a Novel E2F Target Gene Regulated by Transforming Growth Factor-β

**DOI:** 10.1371/journal.pone.0013941

**Published:** 2010-11-10

**Authors:** David Judah, Wing Y. Chang, Lina Dagnino

**Affiliations:** 1 Department of Physiology and Pharmacology, Children Health Research Institute and Lawson Health Research Institute, University of Western Ontario, London, Canada; 2 Department of Paediatrics, Children Health Research Institute and Lawson Health Research Institute, University of Western Ontario, London, Canada; Roswell Park Cancer Institute, United States of America

## Abstract

Regulation of gene expression requires transcription factor binding to specific DNA elements, and a large body of work has focused on the identification of such sequences. However, it is becoming increasingly clear that eukaryotic transcription factors can exhibit widespread, nonfunctional binding to genomic DNA sites. Conversely, some of these proteins, such as E2F, can also modulate gene expression by binding to non-consensus elements. E2F comprises a family of transcription factors that play key roles in a wide variety of cellular functions, including survival, differentiation, activation during tissue regeneration, metabolism, and proliferation. E2F factors bind to the Erb3-binding protein 1 (*EBP1*) promoter in live cells. We now show that E2F binding to the *EBP1* promoter occurs through two tandem DNA elements that do not conform to typical consensus E2F motifs. Exogenously expressed E2F1 activates *EBP1* reporters lacking one, but not both sites, suggesting a degree of redundancy under certain conditions. E2F1 increases the levels of endogenous EBP1 mRNA in breast carcinoma and other transformed cell lines. In contrast, in non-transformed primary epidermal keratinocytes, E2F, together with the retinoblastoma family of proteins, appears to be involved in decreasing EBP1 mRNA abundance in response to growth inhibition by transforming growth factor-β1. Thus, E2F is likely a central coordinator of multiple responses that culminate in regulation of *EBP1* gene expression, and which may vary depending on cell type and context.

## Introduction

The E2F family of transcription factors has frequently been associated with regulation of cell cycle progression from G1 to S phase [1], [2]. However, additional studies, including genome-wide analyses, have revealed that E2F factors modulate a plethora of cellular functions, including DNA replication and repair, apoptosis, signal transduction, and metabolism [1], [2]. Not surprisingly, an increasing number of pathologies have been associated with abnormal E2F expression or activity, ranging from developmental diseases and cancer to neurodegenerative disorders [3], [4].

E2F proteins belong to a class of transcription factors that appear to predominantly bind proximal promoter regions close to the transcription start site of target genes [5]. These interactions can result in stabilization of general transcription machinery and activation of gene expression, or in transcriptional repression, depending on E2F interactions with other proteins, or on the particular E2F member bound to the DNA [6]. Early analysis of E2F-regulated promoters identified DNA sequences that conformed to the consensus 5′-TTTSSCGC- 3′ (where S is either C or G) [7]. However, genome-wide analyses have revealed that only a minority of E2F-bound promoters actually contain this consensus site [8]. Although the functional significance of most of these sites remains to be assessed, regulation by E2F upon binding to a diverse group of sequences, such as 5′-GGGCGGGC-3′ and 5′-GCTCCAAA-3′, has been demonstrated for the ASK-1, and the carboxylesterase gene promoters, respectively [8], [9], [10].

Most high-throughput analyses of putative E2F targets have been conducted using transformed cell lines. In an effort to identify novel E2F targets in normal cell types, we undertook a genome-wide analysis of E2F-bound genes in human epidermal keratinocytes, using chromatin immunoprecipitation (ChIP) coupled to CpG island microarray analysis. We found that E2F1 can bind the ErbB3-binding protein 1 (*EBP1*) promoter in live cells [11].

EBP1 is a ubiquitous, cell cycle-regulated protein, which can suppress growth in certain types of prostate and breast carcinoma cells, and can regulate sensitivity to Tamoxifen in certain breast tumors [12]. EBP1 appears to fulfill multiple diverse biological functions. It can bind DNA, RNA and proteins, and can modulate transcription, protein translation and protein stability [13]. The biological effects of EBP1 vary depending on cell type and context, although the precise mechanisms for the differential roles and regulation of EBP1 in different tissues have not been defined. Significantly, EBP1 can repress E2F transcriptional activity in reporter assays, through its ability to bind to histone deacetylase 2 and pRB [14], suggesting the possibility of cross-talk between the E2F and EBP1 pathways. In this report, we have explored how E2F1 regulates the *EBP1* promoter, and demonstrate the functional importance of dual DNA elements in modulating *EBP1* transcriptional activation by E2F proteins.

## Results

### Identification of *EBP1* as an E2F target gene

**Figure 1:**
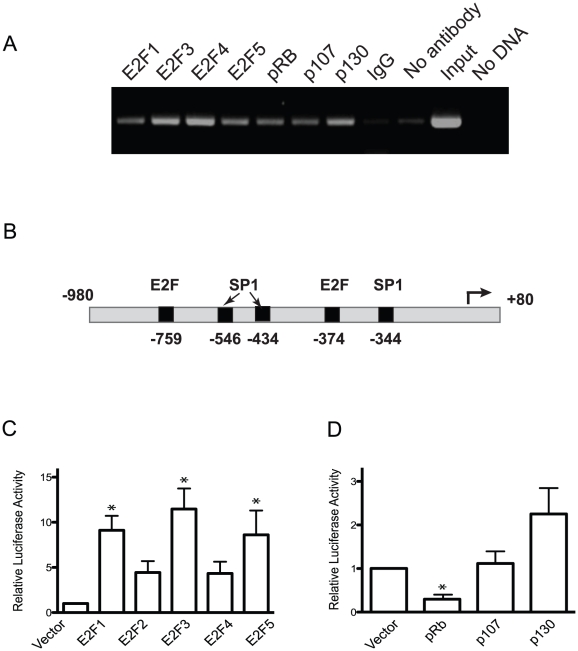
Regulation of the *EBP1* promoter by E2F and pRb proteins. (A) Chromatin immunoprecipitation assays were conducted with antibodies against the indicated proteins and chromatin isolated from exponentially proliferating, unsynchronized cultures of primary epidermal keratinocytes. The DNA immunoprecipitated was amplified with primers specific to the proximal promoter of the *EBP1* gene. Controls included immunoprecipitation with an irrelevant IgG and amplification with 0.2% of the isolated chromatin, prior to immunoprecipitation (‘Input”). (B) Schematic diagram of the human *EBP1* promoter fragment isolated and used in reporter assays. The positions relative to the transcription start site (set to +1) of putative binding sites identified *in silico* for the indicated transcription factors are shown. (C, D) The firefly luciferase reporter driven by the *EBP1* promoter fragment shown in (B) (−980 Luc) was transiently transfected into primary human keratinocytes, together with vectors encoding the indicated E2F or pRb family proteins. All samples were cotransfected with a vector encoding *Renilla* luciferase. *EBP1*-luciferase activity was normalized to *Renilla* luciferase. The results are expressed as the mean +SEM (n = 3) relative to normalized luciferase activity in samples transfected with −980 Luc together with empty vector not encoding for any E2F or pRb protein (set to 1). * indicates p<0.05 (ANOVA).

To determine whether *EBP1* is a *bona fide* E2F target, we first conducted ChIP experiments using chromatin isolated from asynchroous cultures of primary human epidermal keratinocytes. We determined that E2Fs 1, 3, 4, and 5 were capable of binding this promoter (Fig. 1A). We extended this analysis to the retinoblastoma family of proteins (pRb, p107 and p130), as they modulate E2F transcriptional activity, and found that all three proteins associated with the proximal *EBP1* promoter in live keratinocytes (Fig. 1A), which is consistent with our previous reports [11].

To begin to examine the functional significance of E2F binding to the *EBP1* promoter, we used polymerase chain reaction (PCR) to amplify human keratinocyte genomic sequences corresponding to positions −980 to +80 (termed −980 Luc), relative to the predicted transcription start site. *In silico* analyses of this fragment revealed a consensus E2F site centered at position −374 (Fig. S1). Two additional sites with about 90% homology to the E2F consensus were identified, centered at positions −759 and +3 (Fig. S1). Putative binding elements for SP1, Ikaros and Heat shock factor 2 (HSF2) were also localized to positions −344, −141 and −44, respectively (Figs. 1B and S1). The *EBP1* fragment we isolated lacks a TATA box, and exhibits promoter activity, as it is capable of directing expression of a luciferase reporter transfected into neonatal epidermal human keratinocytes (Fig. 1C), and is activated about 10-fold by exogenous expression of E2F1, −3 and −5 in these cells (Fig. 1C). We also observed that exogenous E2F was able to activate this construct 2- and 3-fold, respectively, in HaCaT cells (an immortalized line established from human adult keratinocytes) and IMDF dermal fibroblasts [15] (data not shown), indicating that activation of the *EBP1* promoter by E2F is not cell type-specific. These assays were repeated in the presence of exogenously expressed pRb, p107 or p130. We found that pRb significantly decreased *EBP1* promoter activity (Fig 1D). These data show that the *EBP1* promoter displays characteristics consistent with a *bona fide*, novel E2F target.

### Identification of regions modulated by E2F in the *EBP1* promoter

**Figure 2:**
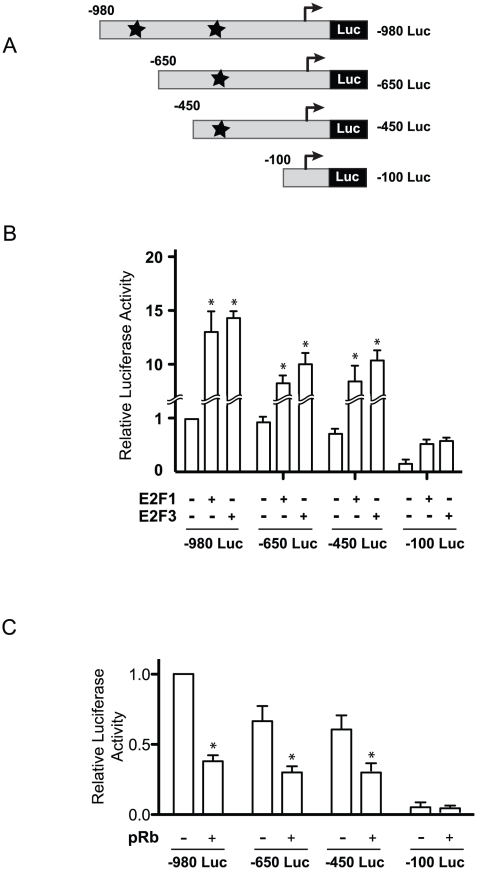
Mapping of E2F- and pRb-responsive regions in the *EBP1* promoter. (A) Schematic of *EBP1* promoter constructs tested. Putative E2F-binding sites at positions −759 and −374 are indicated with a star, and the numbers at right indicate the 5′-flanking sequences present in each construct. (B, C) The indicated *EBP1* constructs were co-transfected with vectors encoding E2F1, E2F3 or pRb into primary human keratinocytes, together with a vector encoding *Renilla* luciferase, used to normalize for transfection efficiency. Normalized luciferase values are expressed as the mean + SEM (n = 3), relative to the basal activity of the −980 Luc construct (set to 1). * indicates p<0.05 (ANOVA).

To identify functionally important *cis-*acting elements in the *EBP1* promoter, we generated a series of reporter constructs containing 5′ deletions, and tested them using luciferase reporter assays (Fig 2A). Deletion of fragments comprising sequences from −980 to −450 was without effect on basal promoter activity, contrasting with the pronounced decrease in transcriptional activity consequent to loss of the region between −450 and −100 (Fig. 2B). To determine the role of this region in E2F-mediated promoter regulation, we measured reporter activity in cells cotransfected with vectors encoding E2F1 or E2F3, as exogenous expression of these two proteins had resulted in pronounced promoter activation (Fig. 1C). Whereas the −980, −650 and −450 to +80 fragments of the *EBP1* promoter were activated to similar extents by E2F1 or E2F3, transcriptional activity of the construct containing the region from −100 to +80 was not significantly altered (Fig 2B). Thus, sequences between positions −450 and −100 are necessary and sufficient for basal and E2F-enhanced transcription. Consistent with this notion, exogenous expression of pRb significantly inhibited promoter activity only in reporters containing the −450 to −100 region (Fig 2C). Additional experiments with promoter fragments containing mutations in one or both of the sites centered at positions −759 and −374 in the context of −980 Luc showed that these alterations had no effect on transcriptional activity (data not shown). Together, these results indicate that the putative E2F sites at −759 and −374 are dispensable for and/or not involved in *EBP1* promoter activity and regulation by E2F.

**Figure 3:**
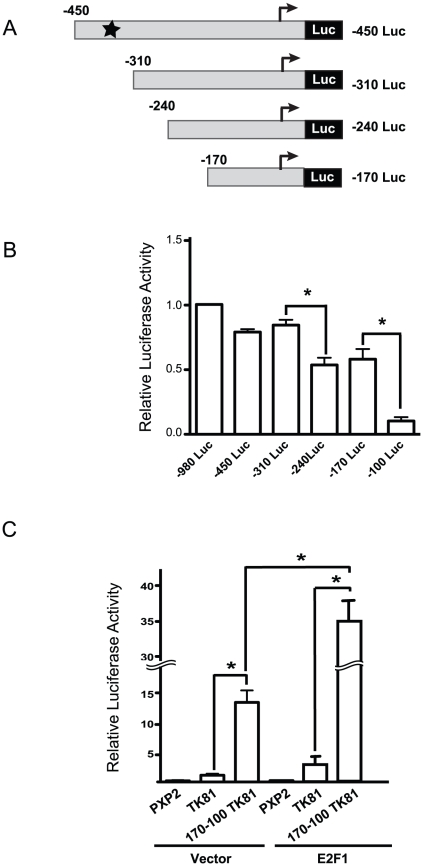
Identification of the E2F response element in the *EBP1* promoter. (A) Schematic of *EBP1* promoter constructs tested. The *in silico* identified E2F-binding site at position −374 is indicated with a star, and the numbers at right indicate the 5′-flanking sequences present in each construct. (B, C) The indicated constructs were transiently transfected together with a plasmid encoding *Renilla* luciferase, used to normalize for transfection efficiency. Normalized luciferase values are expressed as the mean + SEM (n = 3), relative to the basal activity of the −980 Luc construct (panel B) or the TK-81 vector (panel C), set to 1. * indicates p<0.05 (ANOVA). All luciferase values were significantly different from background activity obtained with the empty vector PXP2.

To better map the E2F-responsive region in the *EBP1* promoter, we generated a second series of constructs containing successive 70-bp deletions (Fig 3A). Transient transfection of these constructs in HaCaT cells demonstrated a relatively small decrease in promoter activity with loss of sequences between −310 and −240, and a substantially larger 6-fold reduction with the deletion of sequences between −170 and −100 (Fig 3B). To further confirm that these sequences fulfilled functions as E2F-regulated elements, their ability to activate a heterologous minimal promoter was assessed. We used a modified luciferase reporter containing the minimal promoter of the *Herpes Simplex 1 virus thymidine kinase*. Presence of the −170-to−100 region just upstream from this promoter increased about 12-fold its activity (Fig. 3C). Moreover, exogenous expression of E2F1 induced further activation, as evidenced by an additional 12-fold increase in reporter activity (Fig. 3C). We conclude that an important *cis*-acting region is located within positions −170 to−100 of the *EBP1* promoter.

### Presence of tandem E2F binding sites in the *EBP1* promoter

**Figure 4:**
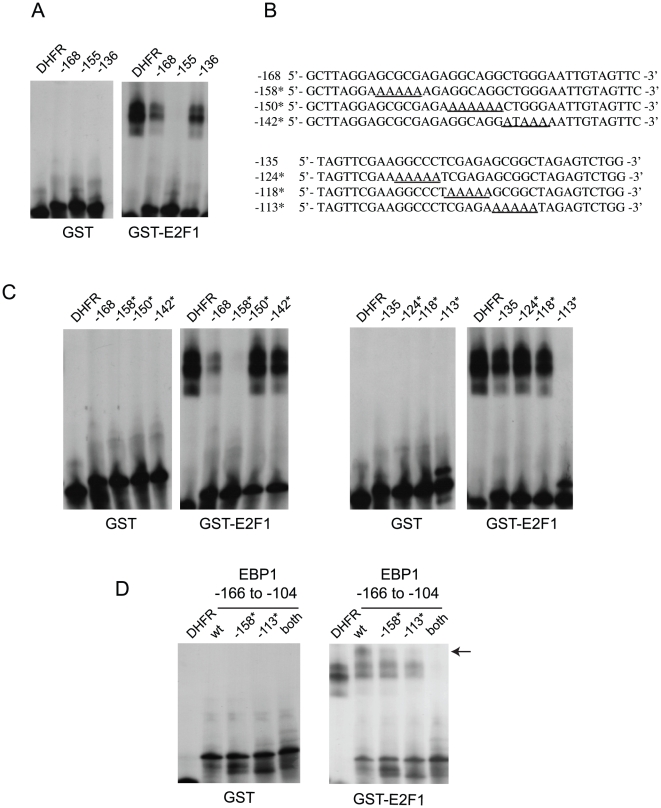
E2F binds to non-consensus tandem sequences in the *EBP1* promoter. (A) Purified recombinant GST or GST-E2F1 was allowed to bind to [^32^P]-labeled, double-stranded oligonucleotide probes corresponding to the following sequences in the *EBP1* promoter: −168 to −130 (−168), −155 to −120 (−155) and −136 to −100 (−136). Protein-DNA complexes were resolved by non-denaturing gel electrophoresis. The lower and the highest mobility signals in the gel correspond, respectively, to GST-E2F1-containing complexes and the free probe. A reaction with a probe corresponding to the E2F- binding site in the dihydrofolate reductase promoter (DHFR) was included as positive control. (B) Sequences of wild type and mutant probes used in EMSA experiments. Numbers on the left indicate the identification number for each probe. Bold, underlined A residues indicate mutations introduced in the probes. (C) EMSA using GST or GST-E2F1 together with a [^32^P]-labeled, double-stranded oligonucleotide probe corresponding to the DHFR E2F-binding site, or one of the sequences shown in panel B, as indicated. (D) EMSA using GST or GST-E2F1 together with a [^32^P]-labeled, double-stranded oligonucleotide probe corresponding to *EBP1* promoter sequences from −166 to −104. The probes contained either wild type sequences (wt), or mutants in the GC-rich regions centered at positions −158, −113 (indicated with *), or both. The arrow indicates a lower mobility complex abundant in the binding reaction containing wild type probe. The highest mobility signals correspond to the free probe.

The results described above indicated the presence of E2F response elements distinct from the putative sites identified *in silico*. As no other obvious consensus E2F sites are present in the region of interest, we conducted gel electropheretic mobility shift assays (EMSA), using probes corresponding to various segments of this promoter fragment (Table S1) and *in vitro* purified recombinant E2F1, bacterially produced as a GST fusion protein. GST-E2F1 proteins have been widely used to characterize E2F binding to DNA *in vitro* [16], and we reasoned that this approach would allow us to define elements in the *EBP1* promoter directly bound by E2F. Binding of GST-E2F1 was observed with oligonucleotide probes comprising sequences −168 to −130, as well as −136 to −100, but not −155 to −120 (Fig. 4A), suggesting the presence of two distinct E2F sites. To identify them, we conducted EMSA experiments with linker scanning mutant probes, in which GC-rich regions, known to be preferentially bound by E2F factors, were individually altered to poly-A stretches (Fig. 4B). Mutation of a GCGCG element centered at position −158 in a probe comprising sequences from −168 to −130 abolished GST-E2F1 binding (Fig. 4B, C). Similarly, mutation of a GCGGC sequence centered at position −113 abrogated GST-E2F1 binding to a probe comprising sequences from −135 to −100 (Fig. 4B, C). To confirm that both of these sequences interact with E2F1, we conducted additional shift assays using longer probes corresponding to positions −166 to −104, including mutant probes in which one or both of the binding sites at −158 and −113 were altered (Fig. 4D). We observed GST-E2F1 binding to probes in which only one site had been mutated, indicating that binding to one site does not depend on interactions with the other site. However, GST-E2F1 binding was abrogated when both sites were absent, consistent with the notion that tandem *bona fide* E2F recognition elements in the *EBP1* promoter are centered at positions −158 and −113, relative to the transcription start site. Further, we also noted that the wild type probe that contained both sites gave rise to an abundant complex with very low mobility (indicated with an arrow, Fig. 4D), possibly due to binding of separate E2F1 proteins to each of the two sites, creating a higher order complex.

**Figure 5:**
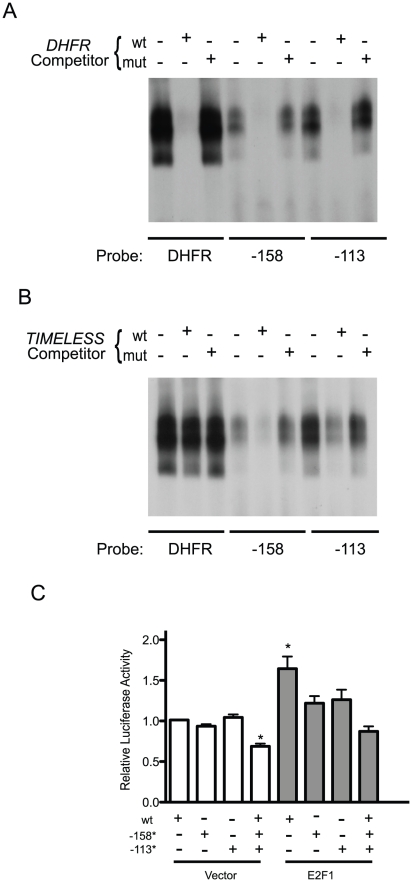
Regulation of the *EBP1* promoter by tandem E2F-binding sites. (A, B) Purified recombinant GST or GST-E2F1 was allowed to bind to [^32^P]-labeled, double-stranded oligonucleotide probes corresponding to the DHFR E2F-binding site, or *EBP1* promoter sequences containing the E2F binding site centered at position −158 or at position −113. Binding reactions were conducted in the presence of a 100-fold molar excess of unlabelled oligonucleotides corresponding to the wild type (wt) or mutant (mut) *DHFR* E2F-binding site (panel A), or wild type (wt) or mutant (mut) E2F-binding site in the *TIMELESS* promoter (panel B). Protein-DNA complexes were resolved by non-denaturing gel electrophoresis. GST-E2F1-containing complexes are shown. (C) Luciferase reporter constructs corresponding to positions −980 to +80 of the *EBP1* promoter were transiently transfected into HaCaT cells, together with a vector or a plasmid encoding E2F1. The EBP1 constructs used were either wild type (wt), or mutants in which the E2F-binding sites centered at positions −158 (−158*) and/or −113 (−113*) were mutated. *EBP1*-directed luciferase activities were normalized to *Renilla* luciferase, and expressed relative to the activity of the wild type −980 Luc construct, which was set to 1. The results are expressed as the average + SEM (n = 3). * indicates p<0.05 (ANOVA) relative to −980 Luc in the absence of exogenous E2F1.

The two E2F sites identified do not conform to classical E2F-binding consensus sequences, such as those found in the *DHFR* promoter. To further investigate the interaction between E2F and the *EBP1* sites, we conducted competition EMSA experiments using other consensus and non-consensus E2F-binding sites. The presence of a 100-fold molar excess of unlabelled oligonucleotides corresponding to either the *DHFR* or the *N-myc* classical E2F-binding site abolished the interaction between GST-E2F1 and both the −158 and the −113 *EBP1* sites (Fig. 5A and data not shown). In contrast, competition with unlabelled oligonucleotides in which the *DHFR* or *N-myc* E2F sites had been mutated to poly-A stretches had no effect on GST-E2F1 binding to the *EBP1* probes (Fig. 5A and data not shown), further confirming the specificity of the interactions between GST-E2F1 and the *EBP1* sites. We next used the E2F site in the *TIMELESS* promoter as a model of a non-consensus E2F site, as its ability to bind E2F has been well established and characterized [8]. The presence of a 100-fold molar excess of wild type, but not mutant, *TIMELESS* promoter sequences substantially reduced binding of GST-E2F1 to each of the *EBP1* sites (Fig. 5B). Unexpectedly, the same probe showed very inefficient competition for the *DHFR* consensus site under the same conditions (Fig. 5B). Recent analyses of genome-wide E2F binding have indicated that E2F target genes can exhibit non-consensus binding sites, which differ from the consensus sequence, exhibit lower affinity for E2F *in vitro*, and may be involved in different modes of E2F binding [8], [9]. Although our studies did not measure relative affinities, they do confirm the specificity of GST-E2F1 binding to each if the *EBP1* elements identified.

### Functional role of the tandem E2F sites in the *EBP1* promoter

To assess the functional role of the E2F binding sites in *EBP1* transcriptional activity, reporter assays were conducted in asynchronous cultures of HaCaT cells, using constructs based on the −980 Luc backbone, as well as −158 and/or −113 mutants with the same alterations shown to abolish GST-E2F1 binding. The basal activity of the wild type and mutant promoter constructs in which one of the two sites was intact was comparable. In contrast, the joint loss of both E2F sites significantly reduced basal reporter activity (Fig. 5C), suggesting that there may be a certain degree of redundancy between these two sites under these conditions. To determine whether these sites are involved in transcriptional activation by E2F, we conducted similar experiments in the presence of exogenously expressed E2F1. The latter significantly increased the activity of the wild type, but not the double mutant promoter (Fig 5C). Unexpectedly, the presence of exogenous E2F1 did not significantly enhance the activity of *EBP1* constructs containing only one wild type E2F site (Fig. 5C). Thus, both sites appear to be necessary for *EBP1* promoter activation under conditions in which E2F1 is overexpressed. Regardless, our data show that the *EBP1* promoter contains two functionally important sites bound and regulated by E2F1. These sites appear to exhibit a certain degree of redundancy under some circumstances.

### Regulation of endogenous *EBP1* expression by E2F

**Figure 6:**
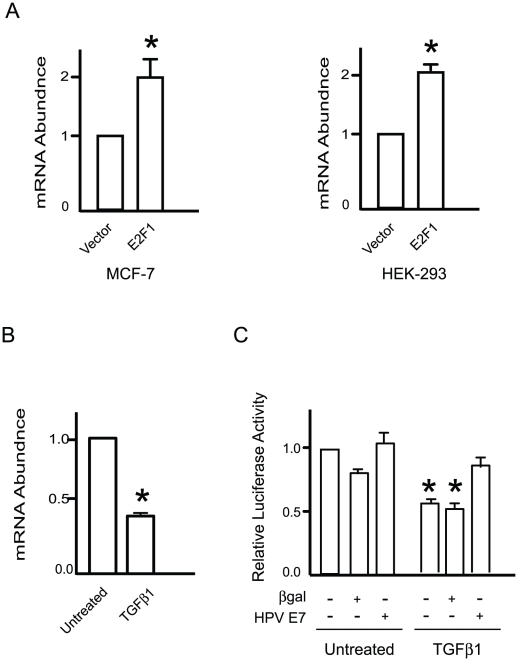
Regulation of *EBP1* gene expression by E2F/pRb. (A) Exponentially proliferating MCF-7 or HEK-293 cells were transiently transfected with an empty vector or an E2F1-encoding plasmid. Twenty-four hours after transfection, RNA was isolated from the cultures, reverse-transcribed and subjected to real-time PCR analysis using EBP1-specific primers. The data are normalized to control RPL 20, RPL 27 and RPL 30 RNA, and are expressed relative to vector-transfected cells, set to 1. Results are expressed as mean + SEM (n = 3). * indicates p<0.05 (Student's t test). (B) Triplicate cultures of exponentially proliferating, unsynchronized primary epidermal human keratinocytes were treated for 48 h in the presence or absence of TGF-β1 (10 ng/ml). Total RNA was isolated, fractionated on agarose gels and transferred to a membrane. Northern blot analysis was conducted with [^32^P]-labelled probes corresponding to the EBP1 mRNA, or to 18S rRNA (as a loading control). The results are expressed as the mean + SEM of normalized EBP1 mRNA levels, with abundance in untreated cells set to 1 (n = 3). * indicates p<0.05 (Student's t test). (C) Primary human epidermal keratinocytes were co-transfected with −980 Luc and a vector encoding *Renilla* luciferase. Five hours after transfection, the cells were incubated with recombinant adenoviruses encoding β-galactosidase (β-gal) or HPV-16 E7 for 3 h. Following a 16-h incubation in normal growth medium, the cells were cultured for an additional 24-h period in the presence or absence of TGF-β1 (10 ng/ml). Luciferase activity in keratinocyte lysates was measured and normalized to *Renilla* luciferase. The results are expressed as the mean + SEM (n = 3), with luciferase activity in uninfected cells cultured without TGF-β1 set to 1. * indicates p<0.05 (ANOVA).

To assess the modulation by E2F of endogenous *EBP1* expression, we transiently transfected MCF-7 and HEK-293 cells with vectors encoding E2F1, and measured EBP1 mRNA abundance 24 h after transfection by quantitative real-time PCR. These two cell lines offer the advantage of transfecting at very high efficiency, providing optimal conditions to detect changes in endogenous *EBP1* gene expression in response to transiently transfected E2F1-encoding vectors. In these experiments, the presence of exogenous E2F1 resulted in a significant, 2-fold increase in EBP1 mRNA in both cell types (Fig. 6A), demonstrating that E2F can upregulate the expression of this gene in different transformed cell types.

In non-transformed cells, pRb forms complexes with E2F and inhibits transcription of various targets, including c-myc, cdc25A and telomerase, in response to physiological signals, such as transforming growth factor-β1 (TGF-β1) stimulation [17], [18], [19]. We previously determined that TGF-β1 treatment of primary epidermal keratinocytes results in changes in the pattern of E2F protein binding to the *EBP1* gene [11]. Hence, we measured the effect of TGF-β1 treatment of these cells on EBP1 mRNA abundance, and determined that the latter decreased by about 2-fold in the presence of this growth factor (Fig. 6B). To investigate if such decrease was due to transcriptional repression, we measured the effect of TGF-β1 treatment on the activity of −980 Luc. The presence of this growth factor reduced reporter activity by about 2-fold (Fig. 6C). To examine if pRb is involved in TGF-β1 repression of *EBP1* promoter activity, we transiently transfected keratinocytes with the −980 Luc reporter, followed by treatment with recombinant adenoviruses encoding either β-galactosidase or the E7 protein encoded by human papilloma virus (HPV) type 16. The E7 protein promotes pRb degradation in keratinocytes, thus allowing activation of E2F targets [20]. Significantly, adenovirus-induced expression of E7 abolished TGF-β1 repression of *EBP1* promoter activity, giving rise to luciferase values indistinguishable from those in untreated cells (Fig. 6C). Together, these observations are consistent with a model in which E2F factors are likely important components in the regulation of *EBP1* expression in a variety of cell types and conditions.

## Discussion

The transcriptional regulation of gene expression plays crucial roles in development and homeostasis of all living organisms. Indeed, alterations in transcription factor activity have been directly linked to hundreds of human diseases [21]. As a result, a large body of work has focused on identifying transcription factor targets. We have combined our previous genome-wide analyses [11] with more in-depth functional studies, identifying the *EBP1* gene as a *bona fide* E2F target. Our studies have defined a mechanism of regulation of *EBP1* promoter activity that involves two tandem E2F-binding *cis* elements located in the proximal promoter region. Significantly, the GC-rich core sequences in these two sites are conserved in the *EBP1* promoters of *Homo sapiens*, *Mus musculus*, *Rattus norvegicus*, *Bos Taurus and Mucaca mulatta*. Both sites differ from the elements we originally identified *in silico*. This is consistent with the reported large fraction of E2F-bound elements in the human genome which do not conform to classical consensus E2F-binding sites, underscoring the limitations of *in silico* analysis, as well as the importance of functional analysis of individual targets. Notably, we found that, under steady-state conditions, the consensus E2F site found in the *DHFR* promoter competed more effectively for binding to recombinant E2F1 than the non-consensus elements in the *EBP1* or the *TIMELESS* promoter. This observation is consistent with previous studies demonstrating that the consensus E2F motif is preferred *in vitro* [22]. E2F binding to DNA *in vivo* is modulated by multiple factors in addition to particular motifs. They include adjacent DNA elements and/or post-translational modifications of E2F protein, which may increase binding affinity, as well as chromatin modifications that would increase accessibility to the DNA and E2F interactions with other transcription factors, which allow the formation of more stable, cooperative multimeric complexes [8]. Unresolved issues for future studies include whether binding of other transcription factors to the *EBP1* promoter aid in the recruitment of E2F proteins.

A number of E2F target genes exhibit a complex promoter structure with two E2F sites, including the E2F1, the p107 and, as we showed, the *EBP1* promoter. Each site can contribute differently to control of the target gene. For example, both activation and repression of p107 expression or p107 reporter plasmids are mainly, but not exclusively, regulated through the distal E2F site [23]. Further, it has been shown that the two sites in the p107 gene fulfill distinct roles, depending on the cell type and biological context. In contrast, neither of the two sites identified in the *EBP1* promoter appears to predominate over the other, although loss of both sites resulted in substantial loss of basal and E2F1-induced promoter activity. These observations would suggest that there might be some functional redundancy between the two sites, at least in the framework of reporter assays. Whether this is also the case in the context of native chromatin, and whether each of these sites plays differential roles in activation vs. repression by endogenous E2F proteins is an area of future important research.

EBP1 is a ubiquitous protein with multiple, complex functions involved in transcription and translation, cell proliferation and survival [24], [25], [26], [27]. The mouse orthologue of EBP1 was first identified by virtue of its regulation through the cell cycle. Its expression increases at the G1/S boundary, and is greatly reduced during S phase or in quiescent cells [28]. This pattern of expression is analogous to that of a large number of E2F-regulated genes. Early work characterized EBP1 as a growth suppressor of prostate and breast carcinoma cell lines that express high Erb2/3 levels [13]. Paradoxically, mice with targeted inactivation of *Ebp1* exhibit reduced overall growth, and cultured *Ebp1*-null mouse embryo fibroblasts fail to proliferate normally [27], further demonstrating that EBP1 functions are cell type- and context-specific.

Given the pleiotropic nature of EBP1 activities, and the multiplicity of mechanisms that operate in transcriptional regulation by E2F, the biological consequences of E2F-mediated EBP1 expression are likely to be very complex. For example, in some breast tumors, there is a loss of E2F1 expression and that of its targets, such as EBP1 and apoptosis-inducing genes [29]. These alterations are thought to constitute a mechanism of growth advantage in these tumor types. We have shown that in MCF-7 breast carcinoma cells, exogenous E2F1 expression increased EBP1 transcript levels. These cells belong to a class of breast tumors that express wild type pRb and p53, and in which enhanced growth occurs, at least in part, through ErbB2/3-dependent mechanisms [29]. Exogenous E2F1 expression in MCF-7 cells also induces growth arrest, without adverse effects on cell viability [29], although the contribution of increased EBP1 in these effects of E2F1 remains unexplored. Thus, modulation of E2F1 activity and/or levels may selectively target certain types of tumors to apoptosis and/or growth arrest, sparing normal cells.

Our studies also suggest the possibility that the consequences of E2F regulation of *EBP1* expression may be different in non-transformed cells. For example, in keratinocytes, the pRb family proteins and E2F1 likely participate in the regulation of EBP1 under certain conditions. In asynchronously growing keratinocyte populations, binding of E2F1, -3, -4 and 5, as well as all pRb family proteins has been detected. TGF-β1 treatment of these cells is associated with loss of detectable E2F1, p107 and p130 binding to the *EBP1* promoter [11], and a concomitant decrease in EBP1 mRNA levels (Fig. 6). The inhibitory effects of TGF-β1 on *EBP1* promoter activity are abrogated by the HPV-16 E7 protein. Although E7 targets all pRb family proteins, its effects on *EBP1* expression may be mediated to a large extent by abolishing pRb-mediated transcriptional repression, given that p107 and p130 do not appear to bind to the promoter in the presence of this growth factor. Based on these observations, we propose a model of *EBP1* transcriptional regulation in keratinocytes in which E2F1 activates *EBP1* transcription in exponentially proliferating cells, whereas reversible growth inhibition and entry into quiescence induced by TGF-β1 induce transcriptional repression mediated, at least in part, by pRb complexes containing other E2F proteins, such as E2F3 and/or E2F4.

In summary, we have demonstrated the presence of tandem non-consensus E2F-binding elements in the *EBP1* promoter, which play key roles in its transcriptional activity. We have also shown that E2F1 can activate *EPB1* expression in a variety of cell types, although the ultimate biological consequences of this regulation are likely to differ between cell types and/or context. A key area for future research will be the investigation of the relative roles that each of the two E2F-binding sites play in transcriptional activation and repression in the context of endogenous *EBP1* gene expression. This will provide crucial insights of how E2F target gene regulation is achieved from genomic information.

## Materials and Methods

### Ethics statement

Primary epidermal human keratinocytes were isolated from neonatal foreskins from anonymous donors. Written informed consent from the donors' guardians was obtained to process the tissue and use the cells. Isolation and culture of these cells was undertaken with approval from The University of Western Ontario Research Ethics Board for Health Sciences Research Involving Human Subjects (Protocol No. 10480E).

### Cell culture, transient transfections and adenovirus infections

Primary epidermal human keratinocytes were isolated from neonatal foreskins, and were cultured as described [11], and used between passages 3–5. IMDF mouse dermal fibroblasts [15], HaCaT, MCF-7 and HEK 293 cells were cultured in DMEM supplemented with 4% fetal bovine serum (FBS). All cells were transiently transfected as described [30]. For experiments using adenovirus-mediated E7 expression, triplicate human keratinocyte cultures were transfected with luciferase reporters for 5 h, followed by infection for 3 h with recombinant adenoviruses encoding green fluorescent protein, together with either the E7 protein from HPV 16 [31] or β-galactosidase [32] as a control. A multiplicity of infection of 50–75 was used. Sixteen hours later, cells were treated with vehicle or with TGF-β1 (10 ng/ml) for 24 h. Cell lysates were prepared and luciferase activity was assayed as described below.

### Plasmids

Fragments of the 5′- flanking sequences of the human *EBP1* gene were obtained by PCR amplification using primers shown in Table S1. Amplicons were cloned into the promoterless firefly luciferase vector PXP2 [33]. The base construct containing 980 bp of 5′-flanking sequences (termed −980 Luc) was used as a template to generate additional promoter fragments by PCR. All promoter sequences generated were verified by dideoxy sequencing. *EBP1* constructs containing mutations at positions −158 and −113 were generated using a Quikchange Mutagenesis kit (Stratagene), following the manufacturer's instructions. To generate heterologous promoter constructs, a fragment containing sequences corresponding to positions −170 to −100 was cloned into pTK81, a vector identical to PXP2, but containing a minimal *Herpes Simplex thymidine kinase* promoter preceding the luciferase encoding region [33].

### Chromatin Immunoprecipitation (ChIP)

For ChIP assays, chromatin was isolated from exponentially proliferating, unsynchronized human keratinocytes, as described [11]. Isolated chromatin was sonicated to produce 500–2000 bp fragments for immunoprecipitation with one of the following antibodies: E2F1 (Upstate, 05-376), E2F3 (Santa Cruz, SC-878), E2F4 (Santa Cruz, SC-366), E2F5 (Santa Cruz, SC-999), pRb (BD Pharmingen, 554136), p107 (Santa Cruz, SC-318X), and p130 (Santa Cruz, SC-317X). Following reversal of cross-linking, the DNA was purified using a polymerase chain reaction (PCR) clean-up kit (Qiagen). DNA samples were amplified by PCR, generating amplicons corresponding to positions −431 to −143, relative to the transcription start site, with the following primers: 5′-CGGTGCGGCCTCCACTCTACTCCAC-3′ and 5′-GCGTGCCTCTCGCGCTCCTAAG-3′

### Electrophoretic mobility shift assays (EMSA)

Electrophoretic mobility shift assays were conducted as described [34], except that reactions contained 24 ng/µl of a double stranded oligonucleotide corresponding to a mutant E2F binding site of the adenovirus *E2* promoter incapable of binding to E2F in place of a mutant *DHFR* oligonucleotide to eliminate non-specific binding to the probe. For these assays, 1.8 µg of purified GST or GST-E2F1 was used per binding reaction. Competition assays contained 100-fold molar excess of appropriate unlabeled oligonucleotide.

### Luciferase reporter assays

Cells cultured in 12-well plates were transfected with either 1 µg/well of luciferase reporter, or 250 ng of luciferase reporter and 1 µg of a vector encoding V5-tagged E2F1 (or pcDNA3.1 plasmid as a control) per well. In all cases, 2 ng of *phRL*-CMV plasmid (Promega) encoding *Renilla* luciferase was included to normalize for transfection efficiency. Transfections were conducted as described [30]. Cells were harvested 24 h after transfection, and luciferase activity in cell lysates was measured with Dual Luciferase Assay Kits (Promega) as per manufacturer's protocol. Reporter activity was normalized to *Renilla* luciferase values. All treatments were done in triplicate, and the data were analyzed by one-way ANOVA with Bonferroni post-hoc test, except in experiments in which E2F-encoding vectors were cotransfected, for which two-way ANOVA with no repeated measures was used. Significance was set at P<0.05.

### RNA analysis

RNA from human keratinocytes (untreated or treated with TGF-β1 for 48 hours) was isolated using the guanidinium isothiocyanate method [35]. 20 µg of each RNA sample was fractionated on a 1.2% agarose gel containing 1% formaldehyde, and transferred onto a Hybond-N membrane (Amersham Biosciences). The resulting blots were hybridized for 20 hours at 42°C in UltraHyb solution (Ambion) to [^32^P]-labelled DNA probes. The probes for human EBP1 and 18S rRNA were generated by PCR amplification of human keratinocyte cDNA. EBP1 mRNA levels were normalized to those of 18S rRNA.

For analysis of E2F1 effects on EBP1 expression in MCF-7 and HEK-293 cells, cultures were transfected with a plasmid encoding V5-tagged E2F1, or with empty vector control. Twenty-four hours after transfection, total RNA was isolated using RNeasy Mini kits (Qiagen), as per manufacturer's protocol. Following verification of their integrity, RNA samples were reverse transcribed with Superscript II (Invitrogen), following manufacturer's instructions. The primers used to determine relative transcript abundance were EBP1 and, to normalize, RPL 22, RPL 27 and RPL 30 [36] (Table S1). Triplicate cDNA samples were amplified for 40 cycles (95°C for 15 sec; 50°C for 1 min) and C_t_ values were obtained. The results were analyzed using SDS 2.3 software (Applied Biosystems Inc.). Relative EBP1 transcript levels were calculated using the ΔΔCt method.

## Supporting Information

Figure S1 Sequence of the human *EBP1* promoter. Numbers on the right indicate positions relative to the predicted transcription start site (set to +1, indicated by the arrow). Consensus binding motifs for the indicated transcription factors predicted *in silico* are highlighted and underlined.
(0.03 MB DOC)

Table S1 Primer sequences.
(0.06 MB DOC)
