# Supplementary material for: EBP1 Is a Novel E2F Target Gene Regulated by Transforming Growth Factor-β
Source: PLoS One. 2010 Nov 10;5(11):e13941. doi: 10.1371/journal.pone.0013941 (PMC2978110; doi:10.1371/journal.pone.0013941)
Supplement: Figure S1 — Sequence of the human EBP1 promoter. Numbers on the right indicate positions relative to the predicted transcription start site (set to +1, indicated by the arrow). Consensus binding motifs for the indicated transcription factors predicted in silico are highlighted and underlined. (0.03 MB DOC) [file pone.0013941.s001.doc]

TCTATTTCTTTAGAACAACCATAGCGCATAGTCCTTTTCATTAAGGGTTT -930

TAGTAGGAATCTACAAGGCAACCAATTGGGAATAACAAAAAGAACCTACG -880

TGCTTTAGGACTTATAAAAAGCCCTATAAGCCCTCCTTCAGAGGCCAAAC -830

ACTGAAACCTCCAGATGCTTCTGAATTCATTATCTTAGAAAAGTCATCAA -780

ATCTTTTTATTTTTTCACGGTAAGAACTCTCAACAAACATGTCTTTCTGA -730

*E2F*

ACACTTCCCTTAGGTGCTCCATCCAGGTGCCTGTTATTGGAACAATAAAG -680

TCATGTTACTTCATTAGGAGTCCGGCCTCTAGATTGCGAGGCCTTTAAAT -630

GGATGATCCCTCCGGTGTCTGGCTGCCCAGTTAGCCCCCGTTACCAGCAC -580

CCTTGGTCTTCTTCCACCTGTCTGCCCCTCCCTGTTCTCCCAGCTTCGGA -530

GGACGACTGGACCGGCTGGGCGGGTTTCGCCAGCCGACCCAGGGATCCGA -480

AGAAGGGCGCACCCAGCCTCCCCGACCTAGGTGTAGACACTGCCCACCCG -430

*SP1 SP1*

CTGCGGCTCCACTCTACTCCACCCCTGCCCGCTCGACTTTAAACCTATTT -380

CCCCGCCGTAGCTCCGCCCCTCTCCCCTCAGCCCGCCCCTCTCTGTTACT -330

*E2F SP1*

GGCTCTCGCTCAGCGTTCTCGGTGGAAGTGGTTTTTCCGGGAGAGACCAC -280

GCTTCCCCTCAAGCTCCCCAACGGCTCCGCCTTCCCGCCGGAGCCTGACC -230

CTTCCCAGAGTGCCCGGCGATTCCGGCGTGCGAGGCCCTTGGAGGGCAAG -180

GCCCCAGGGCCTGGCTTAGGAGCGCGAGAGGCAGGCTGGGAATTGTAGTT -130

*IKAROS*

CGAAGGCCCTCGAGAGCGGCTAGAGTCTGGCGGCCGAGAGGACTAGTTGT -80

CCCAGCGTGCCCTGCGCCTCAGCCCGCGCGCTCGCAGCTTCTCGCTCTCG -30

*HSF2*



CCTGCCTGCCCGCTCCCTTGCTTGCTCGCGCTTTCGCTCGCCCTCTCCTC +20

*E2F*

GAGGATCGAGGGGACTCTGACCACAGCCTGTGGCTGGGAAGGGAGACAGA +70

GGCGGCGGC +80

**Figure s1.** Sequence of the human *EBP1* promoter. Numbers on the right indicate positions relative to the predicted transcription start site (set to +1, indicated by ). Consensus binding motifs for the indicated transcription factors predicted *in silico* are highlighted and underlined.
