# Supplementary material for: EBP1 Is a Novel E2F Target Gene Regulated by Transforming Growth Factor-β
Source: PLoS One. 2010 Nov 10;5(11):e13941. doi: 10.1371/journal.pone.0013941 (PMC2978110; doi:10.1371/journal.pone.0013941)
Supplement: Table S1 — Primer sequences. (0.06 MB DOC) [file pone.0013941.s002.doc]

**Table s1.** **Primer sequences**

**A. Primers used to generate 5' deletion and E2F site mutants of the *EBP1* promoter for luciferase reporter assays**

| Primer | Sequence | EBP1 promoter fragment obtained |  |
| --- | --- | --- | --- |
| EBP1 +80 reverse | 5'- CGATAGAGATCTCCCGACATCTTCCTACTACC -3' | -980 to +80 | |
| EBP1 -100 reverse | 5'- CGATGGTACCCTGCCGCCAGACTCTAGCCGCT -3' | -170 to -100 | |
| EBP1 -980 forward | 5'- AGTCAAGCTTAGCGCATAGTCCTTTTCATT -3' | -980 to +80 | |
| EBP1 -795 forward | 5'- AGTCAAGCTTTAGAAAAGTCATCAAATCTT -3' | -795 to +80 | |
| EBP1 -650 forward | 5'- AGTCAAGCTTAGATTGGCGAGGCCTTTAAAT -3' | -650 to +80 | |
| EBP1 -450 forward | 5'- AGTCAAGCTTGTGTAGACACTGCCCACCCG-3' | -450 to +80 | |
| EBP1 -310 forward | 5'- AGTCAAGCTTGGTGGAAGTGGTTTTTCCGG-3' | -310 to +80 | |
| EBP1 -240 forward | 5'- AGTCAAGCTTGAGCCTGACCCTTCCCAGAG-3' | -240 to +80 | |
| EBP1 -170 forward | 5'- AGTCAAGCTTCTGGCTTAGGAGCGCGAGAG-3' | -170 to +80 | |
| EBP1 -100 forward | 5'- AGTCAAGCTTCGGCCGAGAGAGGACTAGTTGT-3' | -100 to +80 | |
|  |  |  | |

**B. Primers used to generate E2F mutant sites centered at –158 or -113**

| Primer | Sequence | E2F site mutated |
| --- | --- | --- |
| EBP1 -158 mut forward | 5'- CAGGGCCTGGCTTAGGA**AAAAA**AGAGGCAGGCT- 3' | -158 |
| EBP1 -158 mut reverse | 5’- AGCCTGCCTCT**TTTTT**TCCTAAGCCAGGCCCTG -3’ | -158 |
| EBP1 -113 mut forward | 5’- GTAGTTCGAAGGCCCTCGAGA**AAAAA**TAGAGTCTGGCGGC -3’ | -113 |
| EBP1 -113 mut reverse | 5’- GCCGCCAGACTCTA**TTTTT**TCTCGAGGGCCTTCGAACTAC -3’ | -113 |

**C. Sequences of wild type and mutant E2F site probes for EMSA***

| Probe | Sequence |
| --- | --- |
| -136 | 5' TAGTTCGAAGGCCCTCGAGAGCGGCTAGAGTCTGG 3'  3' ATCAAGCTTCCGGGAGCTCTCGCCGATCTCAGACG 5' |
| -155 | 5' GAGCTTCGAACTACAATTCCCAGCCTGCCTCTCGCGCTG 3'  3' CTCGAAGCTTGATGTTAAGGGTCGGACGGAGAGCGCGAC 5' |
| -168 | 5' GCTTAGGAGCGCGAGAGGCAGGCTGGGAATTGTAGTTC 3'  3' CGAATCCTCGCGCTCTCCGTCCGACCCTTAACATCAAG 5' |
| -124* | 5' TAGTTCGAA**AAAAA**TCGAGAGCGGCTAGAGTCTGG 3'  3' ATCAAGCTT**TTTTT**AGCTCTCGCCGATCTCAGACG 5' |
| -118* | 5' TAGTTCGAAGGCCCT**AAAAA**GCGGCTAGAGTCTGG 3'  3' ATCAAGCTTCCGGGA**TTTTT**CGCCGATCTCAGACG 5' |
| -113* | 5' TAGTTCGAAGGCCCTCGAGA**AAAAA**TAGAGTCTGG 3'  3' ATCAAGCTTCCGGGAGCTCT**TTTTT**ATCTCAGACG 5' |
| -158* | 5' GCTTAGGA**AAAAA**AGAGGCAGGCTGGGAATTGTAGTTC 3'  3' CGAATCCT**TTTTT**TCTCCGTCCGACCCTTAACATCAAG 5' |
| -150* | 5' GCTTAGGAGCGCGAGA**AAAAAAA**TGGGAATTGTAGTTC 3'  3' CGAATCCTCGCGCTCT**TTTTTTT**ACCCTTAACATCAAG 5' |
| -142* | 5' GCTTAGGAGCGCGAGAGGCAGG**ATAAA**AATTGTAGTTC 3'  3' CGAATCCTCGCGCTCTCCGTCC**TATTT**TTAACATCAAG 5' |
| -166 | 5' TTAGGA**GCGCG**AGAGGCAGGCTGGGAATTGTAGTTCGAAGGCCCTCGAGA**GCGGC**TAGAGTC 3'  3' AATCCT**CGCGC**TCTCCGTCCGACCCTTAACATCAAGCTTCCGGGAGCTCT**CGCCG**ATCTCAG 5' |
| -166 (-158*) | 5' TTAGGA**AAAAA**AGAGGCAGGCTGGGAATTGTAGTTCGAAGGCCCTCGAGA**GCGGC**TAGAGTC 3'  3' AATCCT**TTTTT**TCTCCGTCCGACCCTTAACATCAAGCTTCCGGGAGCTCT**CGCCG**ATCTCAG 5' |
| -166 (-113*) | 5' TTAGGA**GCGCG**AGAGGCAGGCTGGGAATTGTAGTTCGAAGGCCCTCGAGA**AAAAA**TAGAGTC 3'  3' AATCCT**CGCGC**TCTCCGTCCGACCCTTAACATCAAGCTTCCGGGAGCTCT**TTTTT**ATCTCAG 5' |
| -166 (both*) | 5' TTAGGA**AAAAA**AGAGGCAGGCTGGGAATTGTAGTTCGAAGGCCCTCGAGA**AAAAA**TAGAGTC 3'  3' AATCCT**TTTTT**TCTCCGTCCGACCCTTAACATCAAGCTTCCGGGAGCTCT**TTTTT**ATCTCAG 5' |
| DHFR E2F site | 5' CTAGAGCAA**TTTCGCGC**CAAACTTGGATC 3'  3' GATCTCGTT**AAAGCGCG**GTTTGAACCTAG 5' |
| DHFR E2F site (mutant) | 5' CTAGAGCAA**TTTAAAAA**CAAACTTGGATC 3'  3' GATCTCGTT**AAATTTTT**GTTTGAACCTAG 5' |
| N-myc E2F site | 5' GATCCT**TTTGGCGCG**AAAGGCTTAGATC 3'  3' CTAGGA**AAACCGCGC**TTTCCGAATCTAG 5' |
| TIMELESS E2F site | 5' CAGAAAATT**CATAGCGC**TGTGATTTTTAGATC 3'  3' GTCTTTTAA**GTATCGCG**ACACTAAAAATCTAG 5' |
| TIMELESS E2F site (mutant) | 5' CAGAAAATT**CATAAAAA**TGTGATTTTTAGATC 3'  3' GTCTTTTAA**GTATTTTT**ACACTAAAAATCTAG 5' |

* Residues shown in gray are added during radiolabeling. Bases mutated are bold and underlined

**D. Primer sequences for quantitative RT-PCR**

| Primer | Sequence |
| --- | --- |
| Human EBP1 forward | 5'- AGCGACCAGGATTATATTCTCAAG -3' |
| Human EBP1 reverse | 5'- ATAACATCTGCTTTCCTCCCTG -3' |
| Human RPL 22 forward | 5'- TCGCTCACCTCCCTTTCTAA -3' |
| Human RPL 22 reverse | 5'- TCACGGTGATCTTGCTCTTG -3' |
| Human RPL 27 forward | 5'- ATCGCCAAGAGATCAAAGATAA -3' |
| Human RPL 27 reverse | 5'- TCTGAAGACATCCTTATTGAGG -3' |
| Human RPL 30 forward | 5'- ACAGCATGCGGAAAATACTAC -3' |
| Human RPL 30 reverse | 5'- AAAGGAAAATTTTGCAGGTTT -3' |
